# Refractory chronic “ITP”: When platelet size matters

**DOI:** 10.1002/ccr3.1711

**Published:** 2018-07-20

**Authors:** Panagiotis Baliakas, Magdalena Kättström, Maria Rossing, Rose‐Marie Amini

**Affiliations:** ^1^ Department of Clinical Genetics Uppsala University Hospital Uppsala Sweden; ^2^ Science for Life Laboratory Department of Immunology, Genetics and Pathology Uppsala University Uppsala Sweden; ^3^ Section of Hematology Department of Medicine Örebro University Hospital Örebro Sweden; ^4^ Center for Genomic Medicine Copenhagen University Hospital Copenhagen Denmark; ^5^ Department of Immunology, Genetics and Pathology Clinical and Experimental Pathology Uppsala University and Uppsala University Hospital Uppsala Sweden

**Keywords:** ear nose and throat, genetics, hematology, nephrology, pediatrics and adolescent medicine

## Abstract

Inherited conditions associated with thrombocytopenia should be included in the differential diagnosis of young patients with refractory immune thrombocytopenia (ITP), even in the absence of a positive family history. Early identification of such conditions is of vital importance in order to reach the right diagnosis and avoid unnecessary or even harmful medication.

A 19‐year‐old woman with the diagnosis of refractory chronic immune thrombocytopenia (ITP) was referred to the Clinical Genetic department at Uppsala University Hospital. She was diagnosed with ITP at the age of 9 months. At that time point, a bone marrow biopsy (BMB) was performed with the focus being the exclusion of hematological malignancies. No abnormal findings were reported. She was treated with intravenous immunoglobulin (IVIG) infusions and corticosteroids with minimal response. Splenectomy was also proposed but the parents decided to refrain from that option.

At the time of the referral, the patient was not receiving any medication, exhibiting thrombocytopenia grade III and no increased bleeding tendency. No dysmorphic features were noted at examination, while the family history was nonindicative for any inherited hematological condition. A new BMB was performed with no dysplastic features. Inspection of the peripheral smear showed macrothrombocytes (large platelets, Figure 1). No Howell‐Jolly bodies were observed; however, no immunostaining was performed. Blood sample from the patient was analyzed with a targeted panel for hereditary thrombocytopenias. A known recurrent pathogenic variant (c.287C>T, p.Ser96Leu) in the gene for nonmuscle myosin heavy chain IIa (NMMHC‐IIA) was detected, setting the diagnosis of *MYH9*‐related disorder (*MYH9*‐RD). Targeted analysis of the parents was normal suggesting that the mutation had occurred de novo in the index patient. However, gonadal or somatic mosaicism could not be excluded. Re‐examination of the initial peripheral blood smear was able to confirm the presence of macrothrombocytes even at the time of the initial diagnosis of ITP at the age of 9 months.

**Figure 1:**
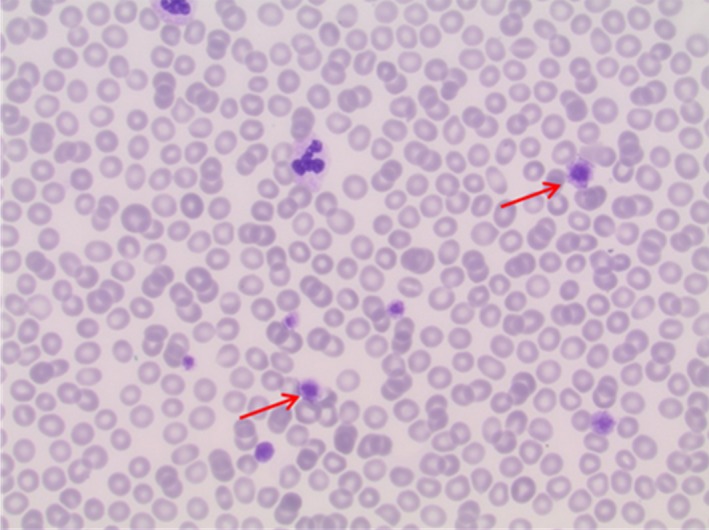
Peripheral blood smear showing macrothrombocytes indicated by the arrows (60× view)

*MYH9*‐related disorder is a well‐described group of conditions where the phenotype includes macrothrombocytopenia in all patients present from birth, as well as a range of manifestations such as hearing loss, cataract, elevated liver enzymes, and renal complications, which may develop anytime between infancy and adulthood.^1^, ^2^, ^3^, ^4^ There is a well‐established genotype‐phenotype association with mutations being observed in the NMMHC‐IIA head domain similar to our patient, predisposing mostly to hearing impairment, while the risk of cataract and kidney failure is significantly lower.^3^ Identification of germline conditions associated with thrombocytopenia is of vital importance in order to reach the right diagnosis and avoid unnecessary or even harmful medication. Moreover, it is essential for planning of eventual surgeries, management of bleeding episodes, as well as inclusion in disease adjusted follow‐up programs. Index patient was referred for audiometric, renal, and ophthalmologic evaluation. A borderline hearing impairment was detected. Of note, cochlear implements have been used with success among patients with *MYH9‐*RD.^5^

Identification of inherited conditions associated with thrombocytopenia should always be included in the differential diagnosis of young individuals with refractory ITP, even in the absence of a positive family history.^6^ Careful assessment of the peripheral smear can be a useful endeavor for the diagnosis of such conditions.

## CONFLICT OF INTEREST

None declared.

## AUTHORSHIP

PB: coordinated the genetic investigation and counseled the family. MK: was the clinical hematologist who referred to the Department of Clinical Genetics. MR: performed the genetic analysis. RMA: inspected the smears of the peripheral blood and the bone marrow.
